# Amyand Hernia Presenting As Acute Appendicitis: A Pediatric Case Report

**DOI:** 10.7759/cureus.109986

**Published:** 2026-05-31

**Authors:** Matthew Daniel, Henry Knox, Shady N Khalil, Saptarshi Biswas

**Affiliations:** 1 General Surgery, Grand Strand Regional Medical Center, Myrtle Beach, USA; 2 Medical Student, Edward Via College of Osteopathic Medicine - Carolinas, Spartanburg, USA; 3 Trauma and Acute Care Surgery, Grand Strand Regional Medical Center, Myrtle Beach, USA

**Keywords:** amyand hernia, appendicitis, inguinal hernia, pediatric inguinal hernia repair, pediatric surgery

## Abstract

Inguinal hernias are among the most common surgeries performed by general surgeons, with more than 20 million repairs occurring globally every year. An Amyand hernia (AH) is a rare atypical inguinal hernia characterized by herniation of the appendix in the inguinal sac. Often occurring in the pediatric population, this type of hernia can often go overlooked due to its presentation that mimics acute appendicitis. Even with a proper work-up, this type of hernia is often particularly challenging to identify, even with ultrasonography or computed tomography. In this report, we present a case of AH that was discovered during an emergent appendectomy for an eight-year-old male patient.

## Introduction

The presence of the appendix within the inguinal canal during appendectomy or inguinal hernia repair is a rare intraoperative finding. This rare entity, an Amyand hernia (AH), is named after Dr. Claudius Amyand, who was the first surgeon to report successful appendectomy during an inguinal hernia surgery [1]. The incidence rates of these sorts of hernia vary widely, with estimates ranging from 0.2% to 1.7% [2]. AH is three times more likely to be diagnosed in the pediatric population due to patency of the processes vaginalis, but it has been reported occurring in patients from three weeks to 92 years of age [1,3]. It most often occurs on the right side of the body but has been reported on the left in cases of situs inversus [1,2].

The presence of appendicitis within an inguinal hernia is even more rare, with an estimated incidence rate of 0.07-0.13% [3,4]. The pathophysiology between AH and acute appendicitis is only partially known at this time [3,5,6]. Some reports have suggested the presence of a patent processus vaginalis as a congenital predisposition between the appendix and the testes [6]. The proposed theories for AH with acute appendicitis are incarceration of the appendix within the hernia sac with subsequent inflammation; inflammation of a prolapsed appendix with venous stasis; irreducibility with bacterial overgrowth and translocation; and development of adhesions between the appendiceal membrane and the hernial sac, resulting in an irreducible hernia [6].

Incidental findings during surgery remain the primary mode of diagnosis for AH. At this time, there is still no true consensus on optimal operative management [3]. While the classification was once considered impractical, it has become important with the use of synthetic mesh materials for repairing inguinal hernias. With the difficulty in preoperative diagnosis of AH, it is important for surgeons to have prior knowledge of this condition to manage it effectively [5]. We present an uncommon case of acute appendicitis that herniated into the right inguinal canal, which was discovered intraoperatively.

## Case presentation

An eight-year-old male patient presented to the emergency department (ED) complaining of right lower quadrant (RLQ) pain that began in the peri-umbilical area 8-12 hours before presentation. The patient endorsed decreased appetite but denied any fever, chills, nausea, vomiting, diarrhea, chest pain, or shortness of breath. The patient had no past medical or surgical history. He was born full term and had an uneventful perinatal course, and the mother had an uncomplicated pregnancy. The patient met all his developmental milestones, took no medications daily, and had no allergies. He also has no known family history of inflammatory bowel disease or gastrointestinal cancer.

During physical examination, the patient was oriented to time, place, and person and appeared to be in no acute distress. He was well nourished and pleasant. The heart displayed a regular rate and rhythm without a murmur. The lungs were clear to auscultation bilaterally with no accessory muscle use. The abdomen had significant RLQ tenderness with a positive McBurney’s point. There was no abdominal rebound tenderness, guarding, or peritoneal signs. A 12-system review was essentially negative except for the abdominal exam. He was afebrile and hemodynamically stable with an elevated white blood cell count of 13.5.

**Figure 1:**
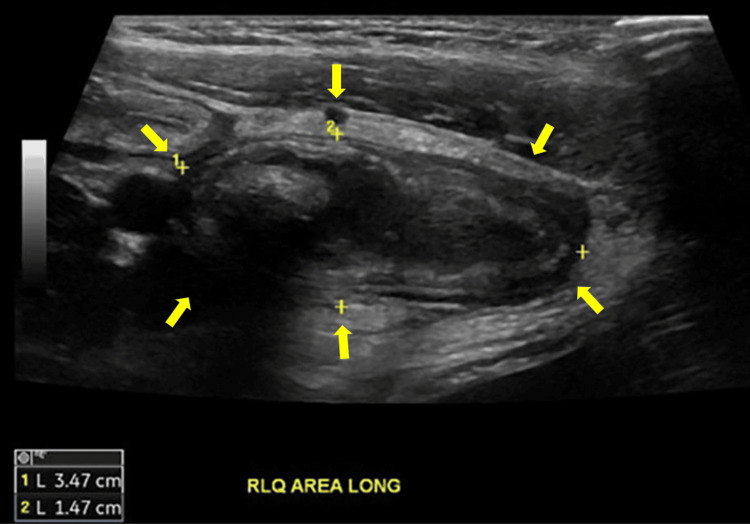
Ultrasound image with arrows pointing to an enlarged appendix with a probable appendicolith.

**Figure 2:**
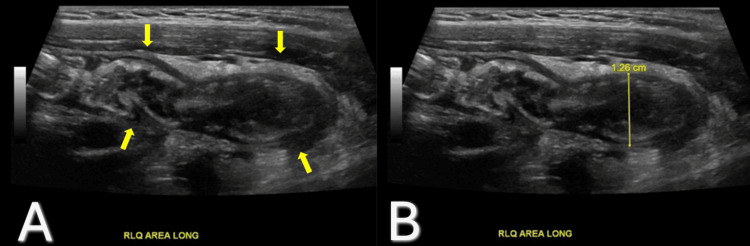
(A) Ultrasound imaging with arrows pointing to a dilated appendix with a (B) maximum diameter of 12.6 mm.

An abdominal ultrasound (US) was completed to assess for potential appendicitis (Figure 1). The US demonstrated an RLQ, non-compressible, dilated, blind-ending tubular structure containing probable stone material without free fluid. These findings were considered strongly concerning for acute calculus appendicitis (Figure 2).

**Figure 3:**
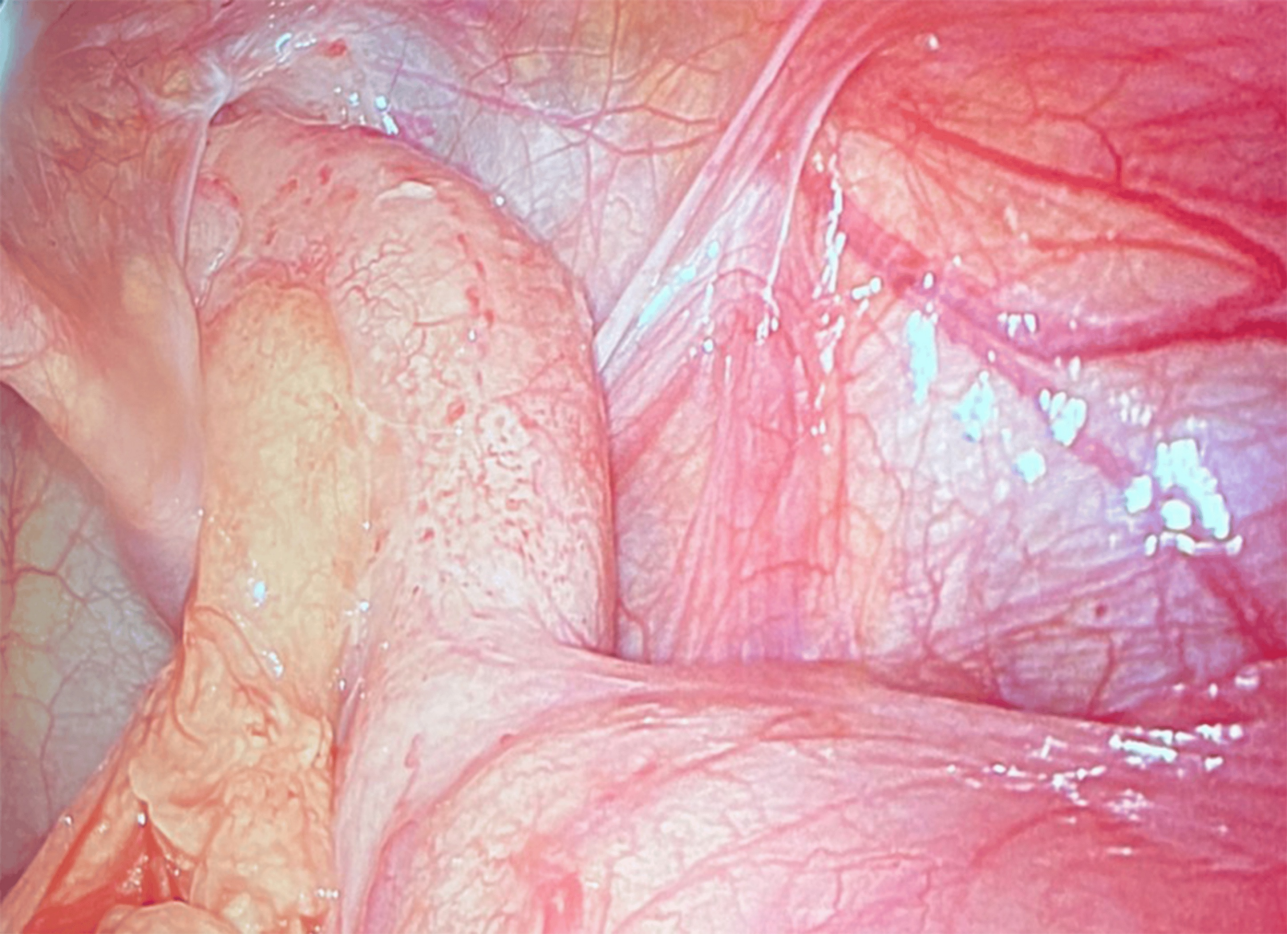
Intraoperative photograph of the patient’s appendix located inside the inguinal hernia.

After discussing the risks and benefits of surgery with the patient and guardian, it was agreed to proceed with a laparoscopic appendectomy. Prior to transport to the operating room (OR), the patient received IV ceftriaxone and metronidazole in the ED. In the OR, he was induced with general anesthesia without any complications. Entry into the abdomen was obtained with an open Hasson technique infra-umbilically. Pneumoperitoneum was induced, and a 5-mm left lower quadrant and a 5-mm right upper quadrant port were placed. The appendix was triangulated. It was large and markedly inflamed with omental adhesions overlying and was found to be located within the inguinal canal (Figure 3). The appendix was grasped and retracted out of the inguinal space. The mesoappendix was removed using a LigaSure device. The abdomen was copiously irrigated with suction. The Hasson port was closed with 0-Vicryl, and the skin was closed with 4-0 Monocryl and finished with Dermabond. The inguinal hernia was not repaired at this time.

The patient tolerated the procedure well and also did well postoperatively. He was tolerating a regular diet, and pain was adequately controlled with an oral regimen. He was discharged home on postoperative day 1. The pathology report demonstrated acute appendicitis.

## Discussion

The probability of an individual having acute appendicitis at some point in their life is approximately 8% [7]. The chances of having an inflamed appendix within an inguinal hernia, an AH, are significantly lower. Almost all AH are diagnosed intraoperatively [7]. Unlike other inguinal hernias that may cause bowel obstruction, an AH usually appears without signs of obstruction or increased inflammatory markers [7]. The differential diagnosis includes strangulated hernia, inguinal hernia, orchitis, appendicitis, peri-appendicular abscess, ileitis, and acute testicular conditions.

Management of AH is still a subject of debate and should be individualized based on a patient’s comorbidities. The most widely accepted classification, which utilizes intra-operative findings, is that of Losanoff and Basson (Table 1) [8]. This classification describes four distinct types. In type I, a normal, non-inflamed appendix is present in the inguinal hernia. In type II, an inflamed appendix is within the inguinal hernia, with no signs of peritonitis or abdominal sepsis. In type III, there is acute appendicitis in an inguinal hernia with associated peritonitis and abdominal sepsis. Lastly, in type IV, there is acute appendicitis in the inguinal hernia with other associated abdominal pathologies [7].

**Table 1:** Losanoff and Basson classification of appendix-containing inguinal hernias and recommended management.

Classification type	Appendix status	Inguinal hernia findings	Peritonitis/Abdominal sepsis	Associated abdominal pathology	Recommended management
Type I	Normal, non-inflamed appendix	Appendix incidentally found within the inguinal hernia	Absent	None	Hernia reduction and mesh repair; appendectomy not routinely recommended
Type II	Inflamed appendix	Acute appendicitis confined to the inguinal hernia	Absent	None	Appendectomy through the hernia sac; primary tissue-based hernia repair (avoid mesh)
Type III	Inflamed appendix	Acute appendicitis within the inguinal hernia	Present	Abdominal sepsis	Appendectomy via laparotomy; management of sepsis; primary hernia repair without mesh
Type IV	Inflamed appendix	Acute appendicitis within the inguinal hernia	May be present	Additional intra-abdominal pathology (malignancy, IBD, perforated viscus)	Appendectomy and hernia repair as indicated; address associated pathology individually
Abbreviations: IBD, inflammatory bowel disease					
Note: Adapted from Losanoff JE, Basson MD. Classification and management of AH [8].					

The optimal treatment should be based on the individual patient and the classification of the hernia. In the literature, there is agreement about the treatment for types III and IV. This includes an appendectomy with a primary hernia repair and avoidance of a mesh [9]. However, there is much less agreement about and increased controversy about the most appropriate treatment for types 1 and II AH [9,10]. In our case, the appendix was inflamed and enlarged within the inguinal hernia, classifying it as type II AH. This required an appendectomy and removal of contents from the inguinal hernia. According to Losanoff’s classifications, type II AH should be treated with an appendectomy and primary closure of the hernia [9].

In this case, a type II AH was discovered, and an appendectomy without closure of the hernia was performed. Although the current literature recommends an appendectomy with primary repair without any mesh, there was no pediatric surgeon available at the time of the discovery of the AH, leading to the decision not to close the hernia. The patient tolerated the procedure well and recovered quickly in the postoperative setting. This case underscores the importance of familiarity with the current literature on AH, as its rare and variable intraoperative findings directly influence operative strategy and postoperative management.

## Conclusions

We present a case of AH, a rare clinical entity, identified intraoperatively as an inflamed appendix within the inguinal canal consistent with a type II classification. Management consisted of appendectomy and primary tension-free hernia repair without mesh, resulting in a favorable postoperative outcome. Although uncommon, AH is most frequently diagnosed intraoperatively, underscoring the importance of surgeon familiarity with established classification systems and management strategies. Ongoing controversy persists regarding the use of prosthetic mesh in type II AH repairs due to concerns of contamination and infectious risk. Further investigation is warranted to refine evidence-based classification frameworks and operative algorithms to optimize surgical decision-making and patient outcomes.

## References

[1] Manatakis DK, Tasis N, Antonopoulou MI (2021). Revisiting Amyand's hernia: a 20-year systematic review. World J Surg.

[2] Feitosa Cavalcante J, Melo Teixeira Batista H, Cavalcante Pita Neto I (2015). Amyand's hernia with appendicitis: a case report and integrative review. Case Rep Surg.

[3] Ivanschuk G, Cesmebasi A, Sorenson EP, Blaak C, Loukas M, Tubbs SR (2014). Amyand's hernia: a review. Med Sci Monit.

[4] Sharma H, Gupta A, Shekhawat NS, Memon B, Memon MA (2007). Amyand's hernia: a report of 18 consecutive patients over a 15-year period. Hernia.

[5] Hatampour K, Zamani A, Asil RS, Ebrahimian M (2023). Amyand's hernia in an elective inguinal hernia repair: a case report. Int J Surg Case Rep.

[6] Jalil S, Azhar M, Malkani I (2023). Amyand's hernia in children. J Pediatric Surg Case Rep.

[7] Kouskos E, Komaitis S, Kouskou M, Despotellis M, Sanidas G (2014). Complicated acute appendicitis within a right inguinal hernia sac (Amyand's hernia): report of a case. Hippokratia.

[8] Losanoff JE, Basson MD (2007). Amyand hernia: what lies beneath--a proposed classification scheme to determine management. Am Surg.

[9] Shaban Y, Elkbuli A, McKenney M, Boneva D (2018). Amyand's hernia: a case report and review of the literature. Int J Surg Case Rep.

[10] Kose E, Sisik A, Hasbahceci M (2017). Mesh inguinal hernia repair and appendectomy in the treatment of Amyand's hernia with non-inflamed appendices. Surg Res Pract.

